# P19 Antibacterial susceptibility patterns and resistance genes from bacterial isolates of neonatal sepsis

**DOI:** 10.1093/jacamr/dlac133.023

**Published:** 2023-01-19

**Authors:** Abumhere S Aziegbemhin, Onaiwu I Enabulele, Faith E Oviasogie, Blessing I Omo-Omorodion, Ikechukwu R Okonkwo

**Affiliations:** Department of Microbiology, Faculty of Life Sciences, P.M.B. 1154, University of Benin, Edo State, Nigeria; Department of Microbiology, Faculty of Life Sciences, P.M.B. 1154, University of Benin, Edo State, Nigeria; Department of Microbiology, Faculty of Life Sciences, P.M.B. 1154, University of Benin, Edo State, Nigeria; School of Health Sciences, Robert Gordon University, Aberdeen, Scotland; Special Baby Care Unit, University of Benin Teaching Hospital, Nigeria

## Abstract

**Background:**

Neonatal sepsis is a bloodstream infection of neonates. It affects newborns globally, particularly in developing countries. Mothers carrying bacterial pathogens may transmit them to their neonates. Poor or inadequate diagnosis often leads to complications with attendant high morbidity and mortality. The need for prompt detection, diagnosis as well as effective treatment monitoring of antibacterial resistant strains is of utmost priority.

**Methods:**

A total of 100 mother-neonate dyad consisting of 90 pairs (with term, preterm or post-term babies) with clinical features of sepsis admitted at the Neonatal Units of Stella Obasanjo Hospital, Central Hospital and University of Benin Teaching Hospital, Benin City and 10 putative controls consisting of apparently healthy neonates were recruited for this study. Bacterial isolation, characterization and identification were carried out using standard microbiology techniques. Antibiotics resistance of bacterial isolates to the routinely used antibacterial agents ampicillin/sulbactam (Unasyn, UN), cefuroxime (CXM), ceftazidime (CTX) and azithromycin (AZM) in neonatal units in this locale was determined using the disc diffusion (Kirby–Bauer) method. Plasmid curing was carried out by inoculating bacteria in 0.1 mL acridine orange. Based on their resistance patterns and curing reaction, one representative bacterial isolates (*Escherichia coli, Pseudomonas aeruginosa, Staphylococcus sciuri* and *Bacillus cereus*) each from saliva, blood and genital swab from five mother-neonate dyad were screened for virulence genes. Antibacterial resistance genes screened for are *mecA*, mph(A) or *bla*_IMP_.

**Results:**

Bacterial isolates showed varying resistance to all antibacterial agents used. Over fifty percent (50%) of the bacterial isolates exhibited MDR phenotype. Unasyn (UN) had more activity agent bacterial isolates. However, resistance to cefuroxime (CXM), ceftazidime (CTX) and azithromycin (AZM) was common. Furthermore, plasmid curing revealed that bacterial isolates harboured resistance plasmid. None of the bacterial isolates were completely cured of their resistant markers. Plasmid-borne antibiotic-resistance markers identified include CTX,UN; CTX,AZM: CXM,AZM; and UN,AZM. Representative bacterial isolates harboured either *mecA* mph(A) or *bla*_*IMP*_ genes.

**Conclusions:**

There is the possibility of transmission of antibacterial resistance genes from bacterial isolates in mothers as well as neonatal intensive care units to neonates thus negatively affecting outcomes of neonatal sepsis and further proliferation of antibacterial resistant strains. Responsible use of antibacterial in this locale is advocated.

